# Exploring the Underdiagnosis and Prevalence of Autism Spectrum Conditions in Beijing

**DOI:** 10.1002/aur.1441

**Published:** 2015-05-06

**Authors:** Xiang Sun, Carrie Allison, Fiona E. Matthews, Zhixiang Zhang, Bonnie Auyeung, Simon Baron‐Cohen, Carol Brayne

**Affiliations:** ^1^ Department of Public Health and Primary CareCambridge Institute of Public HealthUniversity of CambridgeCambridgeUK; ^2^ Autism Research CentreDepartment of PsychiatryUniversity of CambridgeCambridgeUK; ^3^ Cambridgeshire and Peterborough NHS Foundation TrustCambridgeUK; ^4^ MRC Biostatistics UnitCambridge Institute of Public HealthCambridgeUK; ^5^ Pediatrics DepartmentPeking University First HospitalBeijingChina; ^6^ Department of PsychologyUniversity of EdinburghEdinburghUK

**Keywords:** autism, prevalence, screening, diagnosis, prevalence, CAST, China

## Abstract

Previous studies reported that the prevalence of Autism Spectrum Conditions (ASC) in mainland China is much lower than estimates from developed countries (around 1%). The aim of the study is to apply current screening and standardized diagnostic instruments to a Chinese population to establish a prevalence estimate of ASC in an undiagnosed population in mainland China. We followed the design development used previously in the UK published in 2009 by Baron‐Cohen and colleagues. The Mandarin Childhood Autism Spectrum Test (CAST) was validated by screening primary school pupils (n = 737 children age 6–10 years old) in Beijing and by conducting diagnostic assessments using the Autism Diagnostic Observation Schedule and the Autism Diagnostic Interview‐Revised. The prevalence estimate was generated after adjusting and imputing for missing values using the inverse probability weighting. Response was high (97%). Using the UK cutoff (≥15), CAST performance has 84% sensitivity and 96% specificity (95% confidence interval [CI]: 46, 98, and 96, 97, respectively). Six out of 103 children, not previously diagnosed, were found to the meet diagnostic criteria (8.5 after adjustment, 95% CI: 1.6, 15.4). The preliminary prevalence in an undiagnosed primary school population in mainland China was 119 per 10,000 (95% CI: 53, 265). The utility of CAST is acceptable as a screening instrument for ASC in large epidemiological studies in China. Using a comparable method, the preliminary prevalence estimate of ASC in mainland China is similar to that of those from developed countries. ***Autism Res*** *2015, 8: 250–260*. © 2015 The Authors. Autism Research published by Wiley Periodicals, Inc. on behalf of International Society for Autism Research

## Introduction

Autism spectrum conditions (ASC) are characterized by impairments in social interaction and communication, and the presence of repetitive and stereotyped behavior, interests, and activities [World Health Organization, 1993 ]. The *International Classification of Disease, 10th revision* (ICD‐10) [World Health Organization, 1993 ] describes ASC as including four subgroups: childhood autism, atypical autism, Asperger's syndrome (AS) and pervasive developmental disorders—not otherwise specified (PDD‐NOS). Population‐based epidemiological studies in a number of developed countries have reported an increase in the prevalence estimates of ASC, from 30.8 per 10,000 in 2000 [Baron‐Cohen et al., 2000 ] to approximately 100 per 10,000 (1%) [Baron‐Cohen et al., 2009 ]. A recent prevalence estimate of ASC is 113 per 10,000 in the US in 2012 [Centres of Disease Control and Prevention, 2012 ]. One study in South Korea estimated ASC prevalence to be 264 per 10,000 in 2011 [Kim et al., 2011 ]. The most recent worldwide prevalence review suggested the prevalence of ASC is 62 per 10,000 (range 30–116), although the no geographic differences were found among population samples in reviewed studies. [Elsabbagh et al., 2012 ].

Little research has been conducted on the prevalence of ASC in mainland China, whose population now exceeds 1.37 billion. A systematic review on all previous available epidemiological studies in mainland China, Hong Kong, and Taiwan suggested the prevalence of classic autism in China was 11.8 per 10,000 and the prevalence of ASC was 26.6 per 10,000 in Chinese populations, much lower than estimates from developed countries [Sun & Allison, 2009 ]. However, the research methodology adopted in earlier Chinese studies has been different from developed countries, which may have resulted in findings that are not comparable [Sun & Allison, 2009; Tang, Guo, Rice, Wang, & Cubells, 2010 ]. Previous Chinese studies focused mainly on the prevalence of childhood autism (classic autism) and not the whole autism spectrum [Sun, Allison, Matthews, et al., 2013 ]. The recognition of AS or PDD‐NOS subtypes within the autism spectrum is relatively low. Previous Chinese studies have used screening measure that are not designed to detect ASC. The most frequently used *screening* instruments in China have been the Clancy Autism Behaviour Scale [Clancy, Dugdale, & Rendle‐Short, 1969 ] and the Autism Behaviour Checklist (ABC) [Krug, Arick, & Almond, 1980 ], both of which were developed more than three decades ago [The Chinese Autism Society, 2003 ]. Third, there is the lack of standardized diagnostic instruments in Chinese studies. The Childhood Autism Rating Scale (CARS) was used in some of the prevalence studies as a diagnostic instrument [Sun, Allison, Matthews, et al., 2013 ]. However, the diagnosis mainly depended on clinical judgment based on ICD‐10 or the *Diagnostic and Statistical Manual Fourth Edition* (DSM‐IV) [American Psychiatric Association, 1994 ], without the use of any standardized diagnostic instruments. The most frequently used standardized diagnostic instruments in developed countries that were well‐recognized for case identification—the Autism Diagnostic Observation Schedule (ADOS) [Lord, Rutter, DiLavore, & Risi, 2001 ] and Autism Diagnostic Interview‐Revised (ADI‐R) [Rutter, LeCouteur, & Lord, 2003 ]—have not yet been adopted in autism research in mainland China [Sun et al., 2013a ].

The Childhood Autism Spectrum Test (CAST) is a screening instrument designed to identify children with possible ASC, including individuals with classic autism and milder manifestations of autism such as AS. The utility of the CAST has been extensively studied in UK populations. It has not yet been examined cross‐culturally. Due to the stigma related to mental disorders in China, children with classic autism are not usually accepted into mainstream schools in mainland China. [McCabe, 2003; Sun et al., 2012 ] Therefore, potential cases of ASC in mainstream schools would be expected to be children with the “milder” presentations of ASC. A validated screening instrument for ASC including borderline cases is needed to accurately estimate the prevalence of ASC. The aims of the present study were to apply the Mandarin Chinese version of the CAST and international standardized diagnostic instruments to a general population in mainland China for a preliminary prevalence estimation of ASC. The validity of the Mandarin CAST was also reported.

## Method

Ethical approval for this research was sought and obtained from the Peking University First Hospital Ethics Committee and the Cambridge University Psychology Research Ethics Committee.

### The Screening Instrument

The CAST was developed in the UK, specifically for primary school aged children (aged 4–11) because many children with ASC are often not identified prior to attending primary school [Williams, 2003 ]. The CAST is a 37‐item parent‐completed questionnaire, of which 31 items are scored. One point is assigned for an ASC‐positive response and zero for an ASC‐negative response on the scored items. Thus, the total score ranges from 0 to 31 [Baron‐Cohen et al., 2009 ]. Previous studies have demonstrated that using a cutoff of 15, the CAST can be used as a screening instrument in large population‐based epidemiological research for ASC. Using a cutoff of 15, the sensitivity of the CAST is 100%, specificity is 97%, and positive predictive value (PPV) is 50% [Williams et al., 2005 ]. The CAST was used to screen for ASC in a large prevalence study in Cambridgeshire (UK) in 2009 [Baron‐Cohen et al., 2009 ].

### Pilot Sample

A pilot study was conducted to examine whether the UK cutoff of the CAST is suitable to the Chinese CAST. Participants included two groups of children and their parents. Group 1 consisted of 20 children (ages 4–11) with an existing diagnosis of ASC recruited from a database held by the Beijing China Disabled Persons' Federation (BCDPF) and a state‐owned special rehabilitation center (Special Education School of Xicheng District). In order to be registered in the BCDPF, the child must have a confirmed diagnosis of ASC from approved hospitals (well‐recognized hospitals for autism diagnosis nationally) by the BCDPF. Further evaluation and assessments were given to confirm the diagnosis and examine the level of disability. Once the child met all the required criteria on diagnosis and assessment, the child could register with the BCDPF. Group 2 consisted of 20 randomly selected typically developing children from grades 1 to 4 in a mainstream primary school (aged 5–11 years) in the Xicheng District in Beijing.

### Pilot Study Methodology

The CAST was translated from English to Mandarin Chinese by the first author, a native Chinese speaker. The CAST was then translated back into English by two Chinese‐English bilingual speakers, not involved with autism research. In order to be culturally appropriate, language adjustments were made for the translated Mandarin CAST through discussion with a group of professionals specializing in ASC in Beijing. As different Chinese words would be translated into the same English word, the adjustments were made to choose the most appropriate Chinese expression and words that could mostly represent the English version. The resulting version of the CAST was piloted with ten Chinese parents of children between 5 and 10 years of age, opportunistically selected from the outpatients in the Pediatrics Department at Peking University First Hospital (PUFH). The final version of the measure was checked and approved by the authors whose first language was English, following examination of the back‐translated Mandarin CAST. The data of the CAST were entered using Epidata [EpiData Association, 2012; Singh, 2009 ] and analyzed using STATA 10.0 (StataCorp LP, College Station, Texas, USA). The distribution of data was examined using the skewness‐kurtosis test of normality. Differences in score distributions between two groups were investigated using an independent samples *t*‐test. The difference in age between two groups was examined using the two‐sample Kolmogorov–Smirnov test. The association between age and score distribution was examined using linear regression. The UK cutoff of 15 was examined to investigate its applicability to the Chinese population (see Appendix S1).

### Population Screening

A total of 737 pupils in grades 1–4 (aged 6–11) in two mainstream primary schools in Xicheng District in Beijing were invited to participate. The questionnaire packages, including the CAST, information letter, and consent form, were distributed to the parents of these children by class teachers. After a 2‐week interval, the questionnaires and consent forms were collected by class teachers and returned to the first author. Parents were invited using an information letter and were asked to fill in the questionnaire and the consent form. Within the information letter and consent form, the study was introduced as an investigation for social and communicative abilities in children, which will help identify potential problems at school and home. This strategy was used in previous 2009 study in the UK with the purpose of improving participation from families. If the children and their parents were informed the study was of autism or ASC from the very beginning, this could not be comparable to the UK estimate, and also potential stigma toward ASC may influence the completion of the questions and the accuracy of the responses from parents. Participants were excluded if they did not give consent to participate, or if the CAST was returned blank (none were returned blank). A cutoff of ≥15 on the CAST was applied. Missing items were first given an ASC‐negative score (0) to generate a minimum score. The missing items were then given an ASC‐positive score (1) to generate a maximum score. The use of the minimum and maximum scores is explained below (see Statistical Analysis). The screened children were grouped into three bands using their CAST maximum scores in order to capture more potential cases: a high‐score group (≥15) (of which 100% who provided consent were invited to take part in a diagnostic assessment), a borderline group (12–14) (100% who consented were invited to assessment), and a low‐score group (≤11) (5% were randomly selected for assessment). All the assessments were conducted in the Pediatrics Department at PUFH. The randomization of children in the low‐score group was carried out using a random number table.

### Diagnostic Assessment

The ADOS and the ADI‐R were used as diagnostic instruments. Both the Mandarin and Taiwanese versions of the two instruments were provided by the publisher (World Psychological Service, WPS) at the time of this study. After comparison with the original English versions, the Taiwanese versions were translated more accurately, so the Taiwanese versions of the two instruments were used for this study. Due to the possible cultural differences between mainland China and Taiwan, a number of expressions within the Taiwanese versions of ADOS and ADI‐R are not entirely applicable to mainland China. These differences were observed and identified, and all feedback was submitted to the WPS to update the Mandarin versions of the two instruments. The Raven's Progressive Matrices (RPM) is frequently used in clinical neuropsychology for assessing IQ [Raven, 1938 ]. The Chinese version of the RPM was used, which is a validated measure and applicable to individuals from the age of 5 to 75 in mainland China [Li, 1989 ]. Prior to each assessment, parents were asked to provide consent for the assessment to take place, and for the ADOS to be video‐recorded and the ADI‐R to be tape‐recorded. Assessments were conducted by the first author, a medical doctor trained in western and traditional medicine, and also trained in the administration of the ADOS and ADI‐R (by UK trainers). During assessments, the examiner reported and discussed the assessment results with UK experienced trainers and examiners through weekly video meetings and through e‐mails. For suspected ASC cases, the records of assessments were further reviewed and discussed between the examiner and the UK examiners. If there were conflicts between the assessment results obtained from the ADOS and ADI‐R, the child was further examined by experienced child psychiatrist at PUFH. All the children who met diagnostic cutoff on the ADOS or/and ADI‐R were further examined by the child psychiatrist. The final research diagnoses were made following consensus diagnostic discussions with a Chinese child psychiatrist in PUFH.

### Case Definition

Cases of ASC were defined using a consensus case definition. If the child scored above the cutoffs for autism or autism spectrum on both the ADOS and the ADI‐R, a research diagnosis of ASC was made. In this study, if the child scored on both the ADOS and the ADI‐R, or if the child scored above the diagnostic algorithm threshold on either the ADOS or the ADI‐R, he or she was referred to a clinical child psychiatrist at PUFH. The child psychiatrist used all information available from the assessment together with clinical judgment and consultation with DSM‐IV diagnostic criteria. A consensus diagnosis was made after discussion between the Chinese child psychiatrist and the assessment examiner (the first author).

Following the diagnostic assessment, a summary report was provided to each family giving general feedback about the child and a general summary report was given to primary schools. Thus, both parents and teachers would obtain the information on the assessment of the children. At the end of each ADI‐R assessments, the researcher had a 15‐min conversation with the parent to help the parent identify any developmental problems and asked questions regarding possible issues with the child. If there were needs asked by the parent for further referral, another appointment with pediatricians and child psychiatrist was arranged for the families. When the researcher had concerns about a child's development, a recommendation was made at the end of the summary report to parents. Regarding concerns arising from the families who participated in screening but not invited for further assessment, contact information for the PUFH was provided in the invitation letter. Parents with questions contacted the research group at PUFH, and a further appointment with the researcher and pediatrician was arranged and a 15‐ to 20‐min consultation was given to such families to help with their enquiry.

### Statistical Analysis

The normality of score distribution was examined using the skewness‐kurtosis Test. The characteristics of participants who took part in the further assessment and those who refused to participate were compared to assess whether any systematic bias was introduced through nonparticipation in the assessment phase. The characteristics of responders in the low‐score group (≤11) who participated in the further assessment were compared with those of nonparticipants. The Kolmogorov–Smirnov test was used to examine the equality of distributions. The median test was used to investigate whether the two samples were from populations with the same median. The Kruskal–Wallis H test was used to test the difference between medians across multiple groups. Unpaired *t*‐tests and one‐way analyses of variance (ANOVAs) were used to compare means, and chi‐square tests were used to examine differences in proportions. Whenever the numbers were small, a Fisher's exact test was used.

The minimum score was used if there were missing items. Test accuracy of the CAST was examined by calculating the sensitivity, specificity, and PPV using the minimum score. Inverse probability weighting using sampling weights was applied to adjust the estimates for the known nonresponse to the invitation for assessment within each sampling score group [Baron‐Cohen et al., 2009; Williams et al., 2005 ]. This strategy was used because of the two‐phase sampling strategy. The inverse probability was the empirical weight generated according to the response to the screen and to the participation rate in the further assessment phase. A raw prevalence estimate was generated by first using inverse probability weighting. The missing data were then imputed using STATA 10.0, and an adjusted prevalence was provided after adjusting for age, sex, and the nonresponse differences. The 95% confidence intervals (CIs) were calculated accordingly by applying the weighed count. A sensitivity analysis was conducted to investigate the effect of missing data on the CAST by rerunning the analysis using the maximum score. If by using the maximum score, a change of score led to a change in the score group (from <12 to ≥12, or <15 to ≥15), the analyses were rerun without those individuals who changed score group to examine stability of results.

## Results

### Applicability of the UK Cutoff for Mandarin CAST

The mean age of children with ASC in Group 1 was 5.4 (range: 4.1–8.7, standard deviation [SD] = 1.4). The mean age of Group 2 was 8.2 years (range: 6.3–10.6, SD = 1.3). There were 15 boys and 5 girls in Group 1, and 13 boys and 7 girls in Group 2. The difference in age between the two groups was significant (Kolmogorov–Smirnov test, *P* < 0.001). Linear regression showed there was no significant difference in the association between age and CAST score in the two groups (*P* = 0.36). The mean score of the CAST in Group 1 was 20.7 (SD = 3.2) ranging from 15 to 26. The mean score in Group 2 was 6.4 (SD = 2.9), ranging from 1 to 11. The score distributions in Group 1 and Group 2 were normal. The independent sample *t*‐test showed there was a significant difference in the mean score of the CAST between the two groups (*t* = 14.9; *P* < 0.001). Results from this study replicated the previous pilot study of the CAST in the UK [Scott, Baron‐Cohen, Bolton, & Brayne, 2002 ]. The CAST was found to distinguish children with a diagnosis of ASC from typically developing children in primary schools. The results indicate that a cutoff of 15 is a suitable preliminary cutoff for the Mandarin CAST. The score distribution of two groups is provided in Appendix S2.

### Screening and Response

A total of 714 questionnaires were returned and were available for analyses. Therefore, the response rate of screening was 97%. None of the pupils had a previous diagnosis of ASC. Of these, 371 (52.0%) were for boys and 330 (46.2%) were for girls, and for 13 (1.8%), gender was missing. Of the 714 CAST questionnaires, 655 (91.7%) were complete. Fifty‐three (7.4%) had one or two missing items, and six (0.8%) had three to seven items missing.

Date of birth was provided for 687 children. The mean age of the sample was 8.4 years old (SD = 1.2). Of the participants, 544 (76.2%) children were the only child in the family, and 124 children had a brother or sister (17.4%). Information on siblings was missing for 46 (6.4%) children. The distribution of age and sex is shown in Table 1. The occupation and education levels of the parents were also collected and divided into five categories. According to the statistics of educational background in Beijing from the National Bureau of Statistics, the educational level of the parents in this sample is higher than the average in Beijing [National Bureau of Statistics of China, 2012, 2011 ]. The characteristics of the parents are shown in Table 2.

**Table 1:** Age and Sex Distribution of the 714 Children

Age	Sex			Total **(%)**
	Boys	Girls	Missing	
6	66	68	0	134 (18.8)
7	61	52	0	113 (15.8)
8	100	95	2	197 (27.6)
9	105	84	0	189 (26.5)
10	28	23	0	51 (7.1)
11	2	1	0	3 (0.4)
Missing	9	7	11	27 (3.8)
Total	371	330	13	714 (100)

**Table 2:** Characteristics of the Parents

	Category	Number (%)
Father's occupation	Worker or farmer	121 (17.0)
	Clerk	211 (29.6)
	Technical staff	153 (21.4)
	Manager	31 (4.3)
	Own business	119 (16.7)
	Missing	79 (11.1)
Mother's occupation	Worker or farmer	178 (24.9)
	Clerk	165 (23.1)
	Technical staff	168 (23.5)
	Manager	11 (1.5)
	Own business	118 (16.5)
	Missing	74 (10.4)
Father's education	Junior high school	113 (15.8)
	High school	182 (25.5)
	College	316 (44.2)
	Master's or higher	49 (6.9)
	Missing	54 (7.6)
Mother's education	Junior high school	130 (18.2)
	High school	197 (27.6)
	College	308 (43.1)
	Master's or higher	32 (4.5)
	Missing	47 (6.6)

### Assessment Results

**Figure 1:**
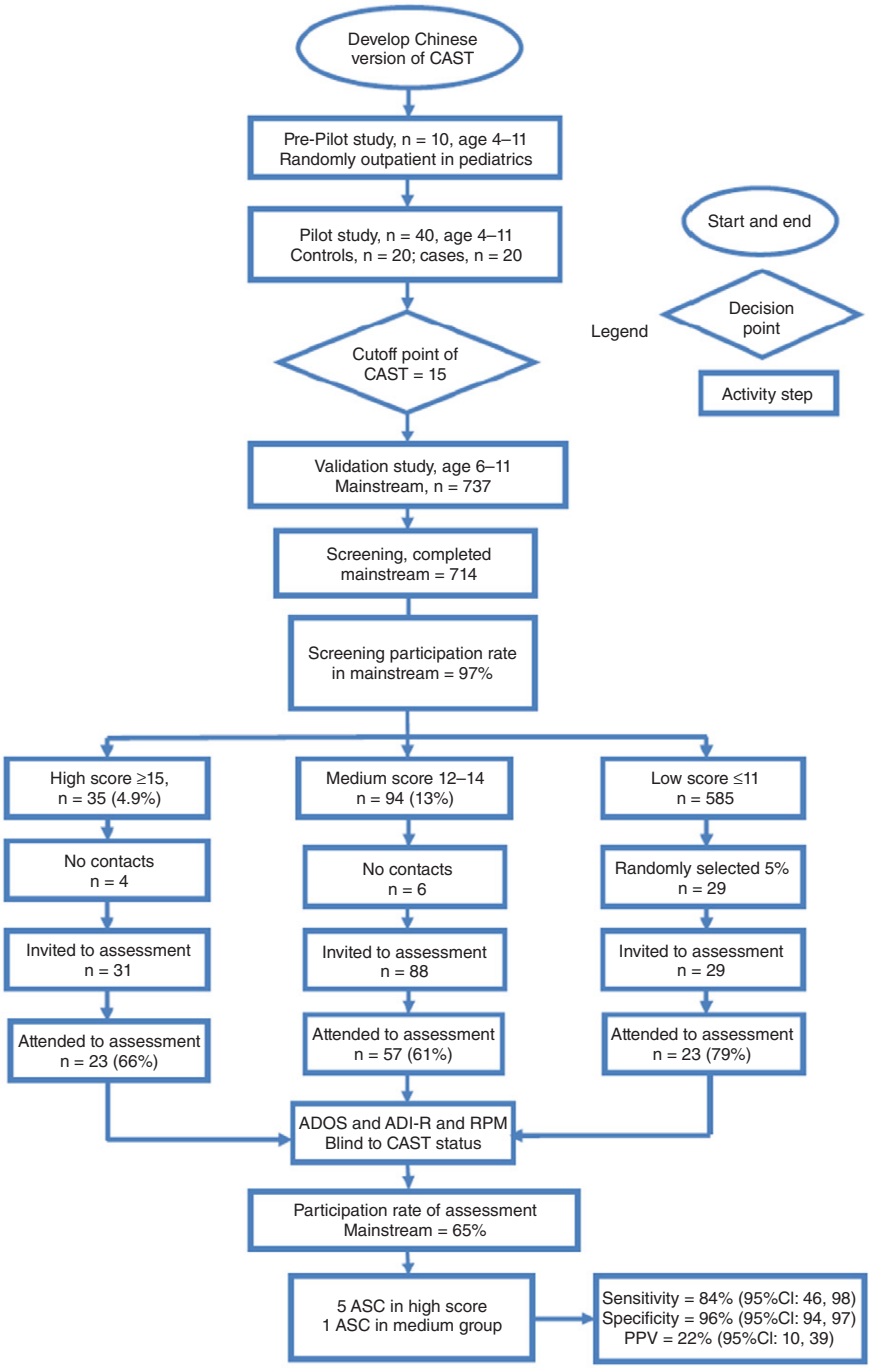
Flowchart of pilot and validation study of the Mandarin CAST. The description of the process of pilot and validation study including results in each step. ADI‐R, Autism Diagnostic Interview‐Revised; ADOS, Autism Diagnostic Observation Schedule; ASC, autism spectrum conditions; CAST, Childhood Autism Spectrum Test; CI, confidence interval; PPV, positive predictive value; RPM, Raven's Progressive Matrices.

The median score on the CAST was 7 (interquartile range: 5, 10; range: 0, 21). The distribution was positively skewed (skewness‐kurtosis test: *P* < 0.005). The maximum score was used to assign the screened children to three scoring groups. Of the 714 screened children, 35 (4.9%) were in the high‐score group (≥15), 94 (13.2%) were in the borderline group (12–14), and 585 (81.9%) were in the low‐score group (≤11). After excluding 10 children with no contact information, 148 children and their families were invited to assessment, and 103 children completed the assessment (participation rate = 65%). The process of the pilot and the main study are shown in Figure 1.

The characteristics of those who completed assessment and those who did not participate are shown in Table 3. Children in the high‐score group who completed the assessment were found to be slightly younger (4 months) than those who were invited but did not participate (unpaired *t*‐test, *P* = 0.01). The mother's education level in the low‐score group who were not invited was found to be lower than that of the other two groups (Fisher's exact test, *P* = 0.039). No other differences between those assessed and those who refused to participate were significant. In the low‐score group (≤11), no significant differences were found between children who were invited and completed the assessment, vs. those who were not invited for an assessment, or vs. those who refused to participate (one‐way ANOVA, *P* > 0.05).

**Table 3:** Comparison of the Characteristics of Participants According to Assessment Status in Different Score Groups

Total	n (%)	Group 1: CAST ≤11			Group 2: 12–14		Group 3: ≥15	
		Invited	Not invited	Invited but no consent	Invited	Invited but no consent	Invited	Invited but no consent
CAST score	Median (IQR)	6 (3,9)	7 (5,9)	7 (6,8)	13 (12,14)	12 (12,13)	16 (15,17)	16 (16,18.5)
Age	Mean (SD)	8.4 (1.2)	8.4 (1.2)	8.6 (1.1)	8.5 (1.2)	8.5 (1.4)	8.1 (1.1)	8.4 (1.6)
Sex								
Boys	n (%)	8 (35)	280 (51)	4 (67)	38 (67)	21 (60)	38 (67)	21 (60)
Girls		15 (65)	267 (49)	2 (33)	19 (33)	14 (40)	19 (33)	14 (40)
Single child								
Yes	n (%)	21 (68)	429 (82)	5 (83)	41 (76)	23 (66)	12 (52)	8 (67)
No		1 (3)	94 (18)	1 (17)	13 (24)	10 (29)	10 (43)	3 (25)
Missing		9 (29)	0	0	0	2 (6)	1 (4)	1 (8)
Father's occupation								
Worker or farmer	n (%)	4 (17)	86 (15)	1 (17)	14 (25)	6 (16)	6 (26)	4 (33)
Clerk		8 (35)	179 (32)	1 (17)	12 (21)	5 (14)	4 (17)	2 (17)
Technical staff		6 (26)	119 (21)	2 (33)	10 (18)	8 (22)	5 (22)	3 (25)
Manager		1 (4)	26 (5)	1 (17)	2 (4)	0	0	1 (8)
Own business		3 (13)	90 (16)	1 (17)	13 (23)	8 (22)	3 (13)	1 (8)
Missing		1 (4)	56 (10)	0	6 (11)	10 (27)	5 (22)	1 (8)
Mother's occupation								
Worker or farmer	n (%)	6 (26)	135 (24)	1 (17)	16 (28)	8 (22)	7 (30)	5 (42)
Clerk		4 (17)	133 (24)	1 (17)	13 (23)	10 (27)	3 (13)	1 (8)
Technical staff		7 (30)	137 (25)	1 (17)	9 (18)	5 (14)	6 (26)	3 (25)
Manager		1 (4)	9 (16)	0	0	0	0	1 (8)
Own business		4 (17)	86 (15)	2 (33)	14 (25)	7 (19)	4 (17)	1 (8)
Missing		1 (4)	56 (10)	1 (17)	5 (9)	7 (19)	3 (13)	1 (8)
Father's education								
Junior high or lower	n (%)	1 (4)	84 (15)	1 (17)	14 (25)	4 (11)	4 (17)	5 (42)
Senor high		6 (26)	143 (26)	2 (33)	14 (25)	10 (27)	5 (22)	2 (17)
College		14 (61)	256 (46)	2 (33)	21 (37)	10 (27)	10 (43)	3 (25)
Graduate		1 (4)	39 (7)	1 (17)	2 (4)	2 (5)	2 (9)	2 (17)
Missing		1 (4)	34 (6)	0	6 (11)	11 (30)	2 (9)	0
Mother's education								
Junior high or lower	n (%)	0	102 (18)	1 (17)	13 (12)	6 (9)	5 (22)	3 (25)
Senor high		9 (7)	145 (26)	3 (50)	20 (18)	11 (17)	5 (22)	4 (33)
College		112 (91)	252 (45)	1 (17)	17 (15)	11 (17)	10 (43)	5 (42)
Graduate		1 (1)	26 (5)	1 (17)	3 (3)	0	1 (4)	0
Missing		1 (1)	31 (6)	0	57 (52)	37 (57)	2 (9)	0

CAST, Childhood Autism Spectrum Test; IQR, interquartile range; SD, standard deviation.

During the diagnostic phase, the examiner was blind to the CAST status. Three children in the high‐score group met cutoffs on both the ADOS and ADI‐R algorithm. Four children met the cutoff of ASC or autism on either the ADOS or ADI‐R. The child who only met the ADI‐R cutoff only missed the ADOS cutoff by 1 point. The two children who met the ADOS diagnosis of ASC missed the cutoff of qualitative abnormalities in communication and repetitive behaviors on the ADI‐R. In total, all seven children were examined by the child psychiatrist. After the diagnosis, those children who presented conflicting results and who scored in high or borderline groups were all given a consensus diagnosis of ASC. One child in the low‐score group did not engage in a few activities during the ADOS assessment. There were limited responses during the interaction that led to a high score on the social and communication scale of the ADOS. The child did not meet the cutoff on the ADI‐R. Moreover, the child behaved cooperatively during the consensus diagnosis with the child psychiatrist. As a result, this child in the low‐score group was given a consensus diagnosis of not having ASC. In total, six cases of ASC were identified during the assessment phase. The parents and teachers of these six children were contacted after the study. Since the grades of these six children were in the average range, with some showing good performance at school, their difficulties in social and communication were not considered to be problematic by their parents. Some of the parents did report having difficulties in communicating with their children, and few of them reported good peer relationships. The characteristics of the six children are shown in Table 4. The results of the ADOS and the ADI‐R are provided in Appendix S3.

**Table 4:** Characteristics of Participants Meeting Assessment Criteria

Participants	CAST		Previous diagnosis^a^	Assessment^b^		Consensus diagnosis^c^	IQ by RPM
	Observed score	Max score	Y/N	ADOS	ADI‐R		
A	16	16	N	Y	Y	Y	118
B	17	17	N	Y	Y	Y	127
C	15	15	N	Y	Y	Y	117
D	16	16	N	N	Y	Y	119
E	15	15	N	Y	N	Y	105
F	12	12	N	Y	N	Y	114
G	2	2	N	Y	N	N	137

a Previous diagnosis of ASC, at the time of interview.

b Above all cutoff on the assessment instrument.

c Met a research diagnosis of ASC. Y = Yes, N = No.

ADI, Autism Diagnostic Interview; ADOS, Autism Diagnostic Observation Schedule; ASC, autism spectrum conditions; CAST, Childhood Autism Spectrum Test; RPM, Raven's Progressive Matrices.

### Prevalence Estimate

Inverse probability weighting was used to adjust the estimates for the known nonresponse to the invitation for assessment within each score group. Using the weightings of 35/23 (35 children scored ≥15 on the Mandarin CAST and 23 completed the assessment) and 94/57 (94 children scored 12–14 on the Mandarin CAST and 57 completed the assessment), the overall directly observed prevalence estimate for all ASC was 9.3 (95% CI: 1.9, 16.7) new (undiagnosed) cases from the screened population. As the total screened population was 714, the raw prevalence of new undiagnosed ASC in this Chinese sample was 130 per 10,000 (95% CI: 58, 286). After imputing the data for missing values using all the available data and adjusting for age, sex, and nonresponse differences, the number of new cases was 8.5 (95% CI: 1.6, 15.4) and the new undiagnosed ASC prevalence was 119 per 10,000 (95% CI: 53, 265).

### Validity of the CAST

**Figure 2:**
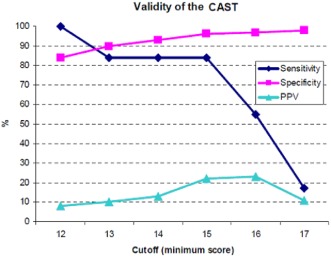
Accuracy indices at each cutoff for a consensus case definition. The sensitivity, specificity, and positive predictive value at each potential cutoff of the Mandarin CAST. At a cutoff of 15, the Mandarin CAST achieved relatively high validity. CAST, Childhood Autism Spectrum Test; PPV, positive predictive value.

Test accuracy was calculated at different cutoffs on the CAST using the minimum score and the consensus diagnosis case definition. At a cutoff of 15, the validity of the CAST was reported the best. At this cutoff, the sensitivity was 84% (95% CI: 46, 98), specificity 96% (95% CI: 94, 97), and PPV 22% (95% CI: 10, 39). When higher cutoffs were used, sensitivity dropped sharply. The highest PPV was 23% (95% CI: 9, 46) at a cutoff of 16. However, sensitivity decreased to 55% at this cutoff (see Fig. 2). A sensitivity analysis using the maximum score revealed the indices of test accuracy to be similar to those of the original calculation using the minimum score. The sensitivity, specificity, and PPV using a cutoff of 15 did not change.

## Discussion

This study suggests that applying international standardized diagnostic instruments and similar epidemiological methods used in developed countries to Chinese primary school age children results in prevalence estimates similar to developed countries. Our results show that using a cutoff of 15, the Mandarin CAST can distinguish children with ASC from typically developing children in China. At this cutoff point, it achieved reasonable sensitivity and specificity as a screening instrument for ASC in primary school‐aged children, but in a low prevalence population its PPV is low, with 78% (18 out of 23) positives proving negative on detailed assessment.

There are several limitations to this study. The study sample was relatively small. The purpose of the pilot was to test the applicability of the CAST in a Chinese population following the methods used in the UK pilot (n = 40) [Scott et al., 2002 ]. The sample for the validation study was drawn from two primary schools, based on resource availability. A large‐scale, population‐based study is needed for further exploration of the prevalence of ASC in China. In addition, Beijing is not a nationally representative region of China in many ways, including socioeconomic status [National Bureau of Statistics of China, 2012, 2011 ]. The results from this study may, therefore, not be representative of the whole population. The response rate for the screening was high, but dropped at the next stage. However, the sample size was still limited. Only 66% children who scored ≥15 took part in the assessment phase, potentially affecting PPV [Eriksson & Westerlund, 2011; Williams et al., 2005 ]. Previous research suggested that the occurrence of psychiatric conditions may be underestimated in studies with a large proportion of nonresponders [Lundberg, Damstrom, Hallstrom, & Forsell, 2005 ]. A prevalence study of ASC found an increase in prevalence estimate when adjusting for nonresponders [Posserud, Lundervold, Lie, & Gillberg, 2010 ]. This study showed a decrease in the prevalence after adjustment for the nonresponders. This finding may be partly because the response was better in boys than girls in this sample.

However, 97% of the whole population of the two schools was screened and then 65% in further assessment; such participation rate should have ensured the representativeness of the total population in these two schools.

Only 5% of children in the low‐score group (≤11) were randomly selected for assessment. However, this could not have led to a verification bias [Alonzo, Brinton, Ringham, & Glueck, 2011; Begg & Greenes, 1983 ]. In this study, it is unlikely that this bias is large as the sampling method for a further assessment was adopted from the UK study of the CAST for the following reasons: (a) in previous UK studies, no case was found in the low‐score group [Baron‐Cohen et al., 2009; Scott et al., 2002; Williams et al., 2005 ]; (b) in the CAST pilot study, noncases scored less than or equal to 11; (c) due to limited resources, it was not possible to assess all children in the low‐score group; and (d) the analysis of those invited and those not invited for an assessment revealed similar sample characteristics. No difference between those invited and not invited in the sample was observed, reducing the likelihood of this bias.

Another limitation of the study is that the ADOS and the ADI‐R have not been previously validated in a mainland Chinese population. However, the Taiwanese versions of both instruments were approved and provided by the publisher (WPS). The differences between the English and Taiwanese versions should not have affected the assessment results. Feedback following this research regarding the administration and translation of these instruments has been submitted to the publisher for updating the instruments.

All ADOS and ADI‐R assessments were conducted by a single researcher over a short time period, with supervision from some of the research team in Cambridge (C.A. and B.A.). Discussions of identified cases with an experienced Chinese child psychiatrist provided robust consensus diagnoses. Such regular consensus‐coding meetings promote reliability throughout the assessment phase.

The study design was adapted from the UK validation study [Williams et al., 2005 ]. The test accuracy indices of the CAST were within the 95% CI of the UK test accuracy indices (sensitivity = 100%, 95% CI: 74, 100; specificity = 97%, 95% CI: 93, 99). However, the proportion of children who scored ≥15 in the UK study (5.8%) was higher than those in the China study (4.9%), while the proportion of children who scored 12–14 in the China study (13.0%) was 2.7 times higher than that in the UK study (4.8%). These differences in score distributions might be due to the subtle differences between the two studies. First, differences in geographical characteristics of samples may lead to differences in score distributions. Second, response to the screening phase in the China study (97%) was much higher than that of the UK study (26%), and the representativeness of the two samples might be different. Third, it is possible that the meaning of certain items differed over the cultures. This might account for the finding that on certain items Chinese parents provided more ASC‐positive responses, which may contribute to the higher proportion of children who scored 12–14 than in the UK study [Williams et al., 2005 ]. Fourth, the social stigma that accompanies children with psychiatric conditions or neurodevelopmental disabilities has been reported in previous studies in China [Lauber & Roessler, 2007; Mak & Kwok, 2010 ]. It is possible that parents might not want their child to be identified as having difficulties of any kind in order to avoid potential social stigma. If this was the case, it is possible that the CAST was rated by parents conservatively, leading to lower CAST scores in the high‐score group and a larger proportion in borderline group in the current Chinese sample.

Although there may be differences in samples and cultural influences, using a cutoff of 15, the Mandarin CAST showed good sensitivity and specificity as a screening instrument for ASC when using standardized diagnostic instruments and consensus diagnostic methods. However, the PPV is low, which led to the finding that only 5 out of 23 screen‐positives were identified as having ASC, following diagnostic assessments. The PPV is associated with sensitivity, specificity, and prevalence of the condition. When both sensitivity and specificity of a test are high, and if the prevalence of the condition in the study sample that the test is applied to is high, the PPV would be expected to be relatively high. If the same test is applied to a sample in which the condition is rare, PPV will be much lower [O'Toole, 2000 ]. Reviewing available screening instruments for ASC within primary school‐aged children, the following instruments can be identified: (a) CARS; (b) ABC; (c) Gilliam Autism Rating Scale; (d) Autism Spectrum Screening Questionnaire (ASSQ); (e) Social Communication Questionnaire (SCQ); (f) Social Responsiveness Scale; and (g) CAST [Sun, 2012 ] (see Appendix S4). However, except the CAST, only the ASSQ and SCQ have been used in general population screening. The PPV of the ASSQ when used in general population in developed countries was around 36% [Posserud, Lundervold, & Gillberg, 2009 ]. The PPV of the SCQ when used in general population was around 32% [Johnson et al., 2011 ]. Only the ASSQ was validated in Chinese population in mainland China prior to this study; however, it was validated in a clinical sample and the PPV of the Chinese ASSQ was not reported [Guo et al., 2011 ]. In previous studies, the PPV of the UK CAST was found to be 50% [Williams et al., 2005 ]. However, in the UK or the US, it is common for mainstream schools to accept children with a diagnosis of autism, while in this Chinese sample no students had an existing diagnosis of ASC since they might not have been accepted by mainstream schools if they did have a diagnosis. Thus, it is very likely that the prevalence of ASC in this Chinese sample is much lower than those of the samples from developed countries, which could partly explain the low PPV of Chinese CAST. This is the first and preliminary study that conducted the screening in the general population in mainland China, which serves a purpose for further development of instrument and methodology for ASC screening in China. In this study, the CAST has been found to be an acceptable instrument for research into ASC. At present, there will be many false positives when screening for ASC in low prevalence populations. This must be balanced against the perceived evidence of benefits to children and their families who do have the condition and may then receive less support. Thus, such an instrument is not ready for use outside research settings.

The prevalence estimate of this undiagnosed Chinese population suggests that there will be many children on the diagnostic spectrum of ASC in the general population in mainland China who have not been identified as meeting ASC criteria. Academic achievement is usually considered the most important aspect of child development by the parents. Due to the emphasis on academic performance and the relatively low awareness of ASC among the general Chinese public, a child's difficulties in social communication and the development of his/her peer relations are likely to be missed by parents and teachers. In addition, the awareness and knowledge of ASC in the general population is limited. Thus, such autistic features are more likely to be considered to be a personality trait or a unique quality rather than a neurodevelopmental disability. Thus, although these children seem to behave relatively “normal” from Chinese parents' or teachers' perspective, their impairments were identified and confirmed by diagnostic assessments and the clinical diagnosis. These children with ASC in ordinary schools might benefit from tailored help and support. Ideally, harms and benefits of such identification should be tested within a trial framework, with provision of current “best practice” support. As indicated by previous research in Chinese populations, the children with classic autism would be less likely to have entered in ordinary primary schools, but are in special intervention settings or at home [Sun et al., 2013b ]. All the children screened in this study were already in ordinary primary schools. Thus, it is mostly likely that these children are on the milder end on the autism spectrum. Thus, the prevalence estimate reported by this study would be an underestimate of ASC in mainland China. The results reported here contribute to furthering the development of research, healthcare, and education services as well as policy for children with ASC in mainland China. However, due to the small sample and the fact that this is a pilot investigation, a further large population‐based study is still needed.

## Supporting information


**Appendix S1.** English CAST and Mandarin CAST package.
**Appendix S2.** Histogram of score distribution of M‐CAST in cases and controls.
**Appendix S3.** Assessment results of children in primary schools.
**Appendix S4.** Screening instruments for primary school‐aged children in developed countries.
