# Supplementary material for: Exploring the Underdiagnosis and Prevalence of Autism Spectrum Conditions in Beijing
Source: Autism Res. 2015 May 6;8(3):250–60. doi: 10.1002/aur.1441 (PMC4690159; doi:10.1002/aur.1441)
Supplement: Supplementary file 1 — Appendix S1. English CAST and Mandarin CAST package. Appendix S2. Histogram of score distribution of M‐CAST in cases and controls. Appendix S3. Assessment results of children in primary schools. Appendix S4. Screening instruments for primary school‐aged children in developed countries. [file aur0008-0250-sd1.zip › aur1441-sup-0001-Suppfile1.doc.doc]

Supplemental 1: English CAST and Mandarin CAST package

**Mandarin CAST package: Information sheet**

**Mandarin CAST package: Main questionnaire**

**Mandarin CAST package: Consent form**
