# Supplementary material for: Exploring the Underdiagnosis and Prevalence of Autism Spectrum Conditions in Beijing
Source: Autism Res. 2015 May 6;8(3):250–60. doi: 10.1002/aur.1441 (PMC4690159; doi:10.1002/aur.1441)
Supplement: Supplementary file 1 — Appendix S1. English CAST and Mandarin CAST package. Appendix S2. Histogram of score distribution of M‐CAST in cases and controls. Appendix S3. Assessment results of children in primary schools. Appendix S4. Screening instruments for primary school‐aged children in developed countries. [file aur0008-0250-sd1.zip › aur1441-sup-0004-Suppfile4.doc.doc]

Supplemental 4: Screening instruments for primary school aged children in developed countries

| Tool | Year | Author | Sample size | Age at screen | Sample source | Cut-off point | Diagnostic  instrument | Reference diagnostic criteria | Sensitivity | Specificity | PPV |
| --- | --- | --- | --- | --- | --- | --- | --- | --- | --- | --- | --- |
| CARS | 2004 | Rellini^1^ | 65 (54 autism, 9 ASD, 1 ADHD, 1 other disorder) | 1.5-11 | Clinical settings | ≥30 for autism | Diagnostic protocol in hospital unit | DSM-IV | 100% | -- | -- |
|  | 2010 | Chlebowski^2^ | 2-year-old: 376  4-year-old: 230 | 2 and 4 | Part of large screening study in clinical settings | ≥25.5 for autism | ADOS, ADI-R | DSM-IV | 2 year old: 92%  4 year old: 82% | 2 year old: 89%  4 year old: 95% | 94%  97% |
| ABC | 1988 | Volkmar^3^  * | 157  (94 autism, 63 no autism) | -- | Clinical settings | Confirm autism: ≥67  Suspicious autism: 53-66 | -- | DSM-III/DSM-III-R | 57% | 62% | -- |
|  | 1991 | Wadden^4^  * | 123  (67 autism, 56 other developmental disorders | 5-17 | Clinical settings | 1. Volkmar’s criteria  2. cut-off=44  3. 34 item version | -- | DSM-III-R or Clinical diagnosis | 1. 49% 2. 87% 3. 85% | 1. 100% 2. 96% 3. 93% | -- |
|  | 1996 | Nordin^5^  * | Group 1: 99 with learn disability and 184 with motor disability  Group 2: 165 from normal schools | 2-17  7-12 | Clinical settings | Weighted score by Krug : ≥67 | -- | Clinical diagnosis | Group1:  ≥67: 38%  ≥45: 100%  Group 2:  ≥67: 29%  ≥45: 65% | Group1:  ≥67: 97%  ≥45: 93%  Group 2:  ≥67: 99%  ≥45: 96% | -- |
|  | 2004 | Rellini^1^ | 65 (54 autism, 9 ASD, 1 ADHD, 1 other disorder) | 1.5-11 | Clinical settings | ≥53 for autism | Diagnostic protocol in hospital unit | DSM-IV | 54% | -- | -- |
| GARS | 2002 | South^6^ | 119 with ASD | 3-10.5 | Five independent research samples | ≥90 as probably autistic | ADOS, ADI-R  VABS | DSM-IV | 48% | -- | -- |
|  | 2005 | Lecavalier^7^ | 284 children and adolescents | 3-21 | General population (local schools) | ≥90 as probably autistic | GADS, SIB-R | DSM-IV | 38% | -- | -- |
|  | 2008 | Sikora^8^ | 109 with Autism  32 with ASD, 51 non-ASD | 1.3-5.9 | Autism program sample | ≥90 as probably autistic | ADOS,  CBCL | DSM-IV | 53% | 54% | -- |
| ASSQ | 1999 | Ehlers^9^  * | Main sample: 110 clinical referral  Validation sample: 34 AS | 6-17 | Clinical settings | ≥13 parent  ≥19 parent  ≥22 teacher | -- | Clinical diagnosis | 91%  62%  70% | 77%  90%  91% | -- |
|  | 2009 | Posserud^10^ | 9,430 | 7-9 | General population | Combined teacher and parent version:  ≥17 | Kiddie-SADS^11^, WISC-III, DISCO | ICD-10, DSM-IV | 91% | 86% | 36% |
|  |  |  |  |  |  |  |  |  |  |  |  |
|  |  |  |  |  |  |  |  |  |  |  |  |
|  |  |  |  |  |  |  |  |  |  |  |  |
| Tool | Year | Author | Sample size | Age at screen | Sample source | Cut-off point | Diagnostic  instrument | Reference diagnostic criteria | Sensitivity | Specificity | PPV |
|  | 2009 | Mattila^12^ | School students: 4480  AS/autism: 47 | 8  7-12 | General population  outpatients | Combined parent and teacher: 30  Teacher version: 22 | ADOS, ADI-R, WISC-III | ICD-10 | 100%  85% | 73%  69% | 35%  28% |
| SCQ | 2006 | Eave^13^ * | 151 clinical referral and preschool clinic | 5 | Clinical settings | ≥15 | ADOS, ADI-R | DSM-IV | 71%  71% | 62%  53% | -- |
|  | 2007 | Chandler^14^ | 247 from special education  Two samples from general population: 411 and 247 | 9-10 | Special education records  General population | 15-21: moderately high;  ≥22: high | ADOS, ADI-R | ICD-10 | 88% | 72% | 64% |
|  | 2007 | Charman^15^ | 119 | 9-13 | Special education | ≥15 for ASD | ADOS, ADI-R | ICD-10 | 86% | 78% | 74% |
|  | 2010 | Johnson^16^ | 219 | 11 | General population | ≥15 for ASD  ≥14 for ASD | DAWBA | DSM-IV-TR | 82%  91% | 88%  86% | 32%  31% |
|  | 2011 | Schanding Jr^17^ | 3,375:  1,663 ASD,  1,712 siblings | 4-17  ≥4 | Previous autism research sample | Teacher: ≥15  Parent: ≥145 | ADOS, ADI-R | -- | Teacher: 60%  Parent: 75% | Teacher: 95%  Parent: 99% | 94%  99% |
| SRS | 2007 | Charman^15^ | 119 | 9-13 | Special education | ≥75 for ASD | ADOS, ADI-R | ICD-10 | 78% | 67% | 63% |
|  | 2007 | Constantino^18^ | 577: 406 with PDD; 171 without PDD | 4-18 | Clinical settings | ≥60 | ADOS,  ADI-R | DSM-IV | 75% | 96% | -- |
|  | 2011 | Schanding Jr^17^ | 3,375:  1,663 ASD,  1,712 siblings | 4-17  ≥4 | Previous autism research sample | Teacher: ≥60  Parent: ≥75 | ADOS, ADI-R | -- | Teacher: 70%  Parent: 80% | Teacher: 95%  Parent: 99% | 93%  99% |
|  | 2011 | Aldridge^19^ | 48 | 4-15 | Clinical settings | Teacher :51.5;  Parent: 56.5; | ADOS, ADI-R | DSM-IV-TR | Teacher: 97%  Parent: 97% | Teacher: 25%  Parent: 8% | -- |
| CAST | 2002 | Scott^20^ | 1,150 mainstream students | 5-11 | General population | ≥15 | ADOS, ADI-R | DSM-IV or ICD-10 | -- | 98% | -- |
|  | 2005 | Williams^21^ | 1,925 mainstream students | 5-11 | General population | ≥15 | ADOS, ADI-R | ICD-10 | 100% | 97% | 50% |

--: not available. DAWBA: Development and Well Being Assessment Psychiatric interview. GADS: Gilliam Asperger Disorder Scale; SIB-R: Scale of Independent Behaviours-Revised. DISCO: Diagnostic Interview for Social and Communication Disorders; CBCL: Child Behaviour Checklist; Kiddie-SADS: Schedule for affective disorders and schizophrenia for school-age-children present and Lifetime version; WISC-III: Wechsler Intelligence Scale for children (3^rd^ ed.); VABS: Vineland Adaptive Behaviour Scale. *Study details were from previous reviews.

References

1.	Rellini E, Tortolani D, Trillo S, Carbone S, Montecchi F. Childhood Autism Rating Scale (CARS) and Autism Behavior Checklist (ABC) correspondence and conflicts with DSM-IV criteria in diagnosis of autism. *J Autism Dev.Disord.* 2004;34(6):703-708.

2.	Chlebowski C, Green JA, Barton ML, Fein D. Using the childhood autism rating scale to diagnose autism spectrum disorders. *J Autism Dev.Disord.* 2010;40(7):787-799.

3.	Volkmar FR, Cicchetti DV, Dykens E, Sparrow SS, Leckman JF, Cohen DJ. An evaluation of the Autism Behavior Checklist. *J Autism Dev.Disord.* 1988;18(1):81-97.

4.	Wadden NP, Bryson SE, Rodger RS. A closer look at the Autism Behavior Checklist: discriminant validity and factor structure. *J Autism Dev.Disord.* 1991;21(4):529-541.

5.	Nordin V, Gillberg C. Autism spectrum disorders in children with physical or mental disability or both. II: Screening aspects. *Dev.Med Child Neurol.* 1996;38(4):314-324.

6.	South M, Williams BJ, McMahon WM, et al. Utility of the Gilliam Autism Rating Scale in research and clinical populations. *J Autism Dev.Disord.* 2002;32(6):593-599.

7.	Lecavalier L. An evaluation of the Gilliam Autism Rating Scale. *J Autism Dev.Disord.* 2005;35(6):795-805.

8.	Sikora DM, Hall TA, Hartley SL, Gerrard-Morris AE, Cagle S. Does parent report of behavior differ across ADOS-G classifications: analysis of scores from the CBCL and GARS. *J Autism Dev.Disord.* 2008;38(3):440-448.

9.	Ehlers S, Gillberg C, Wing L. A screening questionnaire for Asperger syndrome and other high-functioning autism spectrum disorders in school age children. *Journal of Autism Developmental Disorders.* 1999;29(2):129-141.

10.	Posserud MB, Lundervold AJ, Gillberg C. Validation of the autism spectrum screening questionnaire in a total population sample. *J Autism Dev.Disord.* 2009;39(1):126-134.

11.	Kaufman J, Birmaher B, Brent D, et al. Schedule for Affective Disorders and Schizophrenia for School-Age Children-Present and Lifetime Version (K-SADS-PL): initial reliability and validity data. *J Am Acad.Child Adolesc.Psychiatry.* 1997;36(7):980-988.

12.	Mattila ML, Jussila K, Kuusikko S, et al. When does the Autism Spectrum Screening Questionnaire (ASSQ) predict autism spectrum disorders in primary school-aged children? *Eur Child Adolesc.Psychiatry.* 2009;18(8):499-509.

13.	Eaves LC, Wingert HD, Ho HH, Mickelson EC. Screening for autism spectrum disorders with the social communication questionnaire. *J Dev.Behav Pediatr.* 2006;27(2 Suppl):S95-S103.

14.	Chandler S, Charman T, Baird G, et al. Validation of the social communication questionnaire in a population cohort of children with autism spectrum disorders. *J Am Acad.Child Adolesc.Psychiatry.* 2007;46(10):1324-1332.

15.	Charman T, Baird G, Simonoff E, et al. Efficacy of three screening instruments in the identification of autistic-spectrum disorders. *Br J Psychiatry.* 2007;191:554-559.

16.	Johnson S, Hollis C, Hennessy E, Kochhar P, Wolke D, Marlow N. Screening for autism in preterm children: diagnostic utility of the Social Communication Questionnaire. *Arch Dis Child.* 2011;96(1):73-77.

17.	Schanding GT, Jr., Nowell KP, Goin-Kochel RP. Utility of the Social Communication Questionnaire-Current and Social Responsiveness Scale as Teacher-Report Screening Tools for Autism Spectrum Disorders. *J Autism Dev.Disord.* 2011.

18.	Constantino JN, Lavesser PD, Zhang Y, Abbacchi AM, Gray T, Todd RD. Rapid quantitative assessment of autistic social impairment by classroom teachers. *J Am Acad.Child Adolesc.Psychiatry.* 2007;46(12):1668-1676.

19.	Aldridge FJ, Gibbs VM, Schmidhofer K, Williams M. Investigating the clinical usefulness of the Social Responsiveness Scale (SRS) in a tertiary level, autism spectrum disorder specific assessment clinic. *J Autism Dev Disord.* 2011;42(2):294-300.

20.	Scott FJ, Baron-Cohen S, Bolton P, Brayne C. The CAST (Childhood Asperger Syndrome Test): preliminary development of a UK screen for mainstream primary-school-age children. *Autism.* 0/03// 2002;6(1):9-31.

21.	Williams J, Scott F, Stott C, et al. The CAST (Childhood Asperger Syndrome Test): test accuracy. *Autism.* 0/02// 2005;9(1):45-68.
